# mSphere of Influence: Understanding Virus-Host Interactions Requires a Multifaceted Approach

**DOI:** 10.1128/mSphere.00105-20

**Published:** 2020-03-25

**Authors:** Rory D. de Vries

**Affiliations:** a Department of Viroscience, Erasmus MC, Rotterdam, The Netherlands

**Keywords:** measles virus, pathogenesis, virology, virus-host interactions

## Abstract

Rory de Vries works in the field of viral pathogenesis and focuses on interactions between respiratory viruses (or corresponding vaccines) and the host immune system. In this mSphere of Influence article, he reflects on how the articles “Predominant infection of CD150^+^ lymphocytes and dendritic cells during measles virus infection of macaques” by R. L. de Swart et al. (R. L. de Swart, M. Ludlow, L. de Witte, Y. Yanagi, et al., PLoS Pathog 3:e178, 2007, https://doi.org/10.1371/journal.ppat.0030178) and “Long-term measles-induced immunomodulation increases overall childhood infectious disease mortality” by M. J. Mina et al. (M. J. Mina, C. J. Metcalf, R. L. de Swart, A. D. M. E. Osterhaus, and B. T. Grenfell, Science 348:694–699, 2015, https://doi.org/10.1126/science.aaa3662) made an impact on him. These articles studied interactions between measles virus and the host and influenced him by making two important points. (i) It is crucial to use nonadapted (recombinant) viruses in disease-relevant model systems when studying virus-host interactions. (ii) Studying viral pathogenesis requires a combination of *in vitro*, *ex vivo*, and *in vivo* studies, and a group of researchers with multiple expertises. He learned that only when all these aspects are combined, can one truly answer the question: “How does a virus cause disease?”

## Abstract (precis)

Rory de Vries works in the field of viral pathogenesis and focuses on interactions between respiratory viruses (or corresponding vaccines) and the host immune system. In this mSphere of Influence article, he reflects on how the articles “Predominant infection of CD150^+^ lymphocytes and dendritic cells during measles virus infection of macaques” by R. L. de Swart et al. (R. L. de Swart, M. Ludlow, L. de Witte, Y. Yanagi, et al., PLoS Pathog 3:e178, 2007, https://doi.org/10.1371/journal.ppat.0030178) and “Long-term measles-induced immunomodulation increases overall childhood infectious disease mortality” by M.

## COMMENTARY

In 2007, 1 year before starting my Ph.D. on measles pathogenesis and immune suppression, R. L. de Swart and coworkers published an “illuminating” article on the pathogenesis of measles (1). In this article, nonhuman primates were inoculated with a recombinant wild-type-based measles virus (MeV) expressing a fluorescent reporter protein, and viral tropism and pathogenesis were studied. Interestingly, this article was preceded by an inconsistency in the literature: according to the textbooks, MeV initially infected epithelial cells of the respiratory tract, followed by viremia predominantly mediated by monocytes. However, neither of these cell types expressed the known cellular entry receptor for MeV (at that time): signaling lymphocyte activation molecule (SLAM, CD150) (2). de Swart et al. set out to address this inconsistency by performing *in vivo* studies in the only animal model that fully recapitulates measles in humans, nonhuman primates (3–6).

Before the identification of CD150 as the cellular entry receptor for MeV, the virus was classically isolated on human kidney cells, Vero cells, or chicken embryo fibroblasts, none of which express the receptor for MeV. Forced sequential passaging of MeV over nonsusceptible cells led to laboratory adaptation and attenuation, creating the foundation of the live-attenuated measles virus vaccines that are currently in use (7). It was not all good news though: many scientists started using these laboratory-adapted viruses to study virus-host interactions. Among other things, this led to the initial identification of CD46 as a MeV receptor (8). We now know that CD46 can be used only by laboratory-adapted and vaccine strains of MeV, but not by wild-type strains. Coming back to the de Swart et al. article (1), to accurately study measles pathogenesis, it was crucial that they selected a wild-type-like MeV strain that used the correct entry receptors (9). In addition, the recombinant virus used expressed a fluorescent reporter protein, enhanced green fluorescent protein (EGFP) (10). The selection of this virus was inspired by an article by von Messling et al., who studied the pathogenesis of canine distemper virus by inoculating ferrets with an EGFP-expressing virus (11). The outcome of these studies was truly amazing; both von Messling et al. (11) and de Swart et al. (1) were able to study viral pathogenesis and tropism by visualizing EGFP-positive virus-infected cells and demonstrating virus replication on a macroscopic, microscopic, and single-cell level.

This work influenced me directly, because it made it possible to study one of my major interests: how does MeV interact with the host immune system and cause immune suppression? At this point in time, it was generally accepted that measles causes a transient immune suppression; however, the underlying mechanisms and duration were unclear. I studied the underlying mechanisms *in vitro* in human primary cells and *in vivo* in nonhuman primates and found that MeV mainly targets memory T lymphocytes and B lymphocytes, making the host “forget” previous infections. The laboratory I worked in named this “measles immune amnesia” (12, 13). Shortly after publication of these studies, M. J. Mina and coworkers published an impressive article in Science on the public health consequences of measles immune amnesia (14). He performed a retrospective clinical cohort-based study and proved that the incidence of measles is directly associated with long-term (2 to 3 years after measles) noninfectious disease mortality in children. To me, the article by Mina et al. also showed that only by combining data from *in vitro*, *ex vivo*, *in vivo* models with clinical studies can one truly answer the question “How does a virus cause disease?”

In a broader context, these articles have taught me an incredible amount about studying viral pathogenesis and virus-host interactions. The differences in receptor use between laboratory-adapted and wild-type MeV are a beautiful example of why I stress that scientists should pay careful attention to which viruses they use in their studies. Passaging of viruses on nonnatural target cells leads to adaptation, potentially influencing results obtained from experiments with that passaged virus. Similarly, it has taught me to make rational choices when selecting the cell type or animal model in which I perform experiments. Performing *in vitro* experiments in continuous tumor cells is often easy, because these cells are readily available and easy and cheap to maintain. However, depending on the experimental question, I always consider the influence of the choice of cell type on the results and whether it might be better to perform *in vitro* (or *ex vivo*) experiments in primary cells. I have implemented these lessons in our current studies on virus-host interactions of human respiratory syncytial virus (HRSV), where we make use of recombinant viruses directly based on clinical isolates and study their behavior in respiratory organoids or differentiated primary airway epithelial cells cultured at the air-liquid interphase (15). This all sums up to a quote from Alessandro Sette (La Jolla Institute for Immunology, San Diego, CA, USA) that I picked up while working there as a visiting scientist: “Don’t do the experiments you can do (because of ease), but do the experiments that you should do (using disease-relevant model systems).”

Both articles described here were game-changers in the field of measles research; combined with follow-up studies, they led to revisions of the textbooks. Shaping me as a researcher, this is one of the “life lessons” I have taken from these articles: what you read in textbooks or articles is not right *per se* (or as my Ph.D. supervisor, Albert Osterhaus, used to say, “Expect the unexpected”), and scientists should always remain vigilant and “critical.” If one has a hypothesis that deviates from the current dogma, you should be ambitious and perform the experiments to either prove or disprove that hypothesis with perseverance and indomitable spirit.
